# Revalorization of Microalgae Biomass for Synergistic Interaction and Sustainable Applications: Bioplastic Generation

**DOI:** 10.3390/md20100601

**Published:** 2022-09-25

**Authors:** Itzel Y. López-Pacheco, Laura Isabel Rodas-Zuluaga, Sara P. Cuellar-Bermudez, Enrique Hidalgo-Vázquez, Abraham Molina-Vazquez, Rafael G. Araújo, Manuel Martínez-Ruiz, Sunita Varjani, Damià Barceló, Hafiz M. N. Iqbal, Roberto Parra-Saldívar

**Affiliations:** 1 Tecnologico de Monterrey, School of Engineering and Sciences, Monterrey 64849, Mexico; 2 Tecnologico de Monterrey, Institute of Advanced Materials for Sustainable Manufacturing, Monterrey 64849, Mexico; 3 Laboratory of Aquatic Biology, KU Leuven Campus Kortrijk, 8500 Kortrijk, Belgium; 4 Gujarat Pollution Control Board, Gandhinagar 382010, Gujarat, India; 5 Department of Environmental Chemistry, Institute of Environmental Assessment and Water Research, IDAEA-CSIC, Jordi Girona 18-26, 08034 Barcelona, Spain; 6 Catalan Institute for Water Research (ICRA-CERCA), Parc Científic i Tecnològic de la Universitat de Girona, c/Emili Grahit, 101, Edifici H2O, 17003 Girona, Spain; 7 Sustainability Cluster, School of Engineering, University of Petroleum and Energy Studies, Dehradun 248007, Uttarakhand, India

**Keywords:** microalgae, biomass, bioplastics, polyhydroxyalkanoates, wastewater, environmental impact

## Abstract

Microalgae and cyanobacteria are photosynthetic microorganisms’ sources of renewable biomass that can be used for bioplastic production. These microorganisms have high growth rates, and contrary to other feedstocks, such as land crops, they do not require arable land. In addition, they can be used as feedstock for bioplastic production while not competing with food sources (e.g., corn, wheat, and soy protein). In this study, we review the macromolecules from microalgae and cyanobacteria that can serve for the production of bioplastics, including starch and glycogen, polyhydroxyalkanoates (PHAs), cellulose, polylactic acid (PLA), and triacylglycerols (TAGs). In addition, we focus on the cultivation of microalgae and cyanobacteria for wastewater treatment. This approach would allow reducing nutrient supply for biomass production while treating wastewater. Thus, the combination of wastewater treatment and the production of biomass that can serve as feedstock for bioplastic production is discussed. The comprehensive information provided in this communication would expand the scope of interdisciplinary and translational research.

## 1. Introduction

Plastics are high molecular mass synthetic organic polymers mainly derived from hydrocarbons obtained from fossil fuels, such as crude oil and natural gas. Plastics are used for several purposes, including packaging, which typically is not recycled but ends up as waste. In 2015, it was estimated that of the 6300 million metric tons of plastic generated, around 9% was recycled, 12% incinerated, and 79% deposited in landfills or the natural environment [1,2]. If the current production and waste management continue according to these trends (annual growth rate of plastic production around 8.4%), around 12,000 million metric tons of plastic waste will be deposited in landfills or the natural environment by 2050. Furthermore, it is important to note that plastic degradation can range from 58 to 1200 years [3]. Together with the high rate of plastic use, this fact has caused the accumulation of plastics on the planet to be an environmental problem that requires urgent attention.

Plastic accumulation in the aquatic environment is one issue of emerging concern because of its possibility of being ingested throughout the food web and accumulated by living organisms [4]. To visualize the large consumption of plastics and their global impact, the UN General Assembly has reported that 13 million metric tons of plastic leak into the ocean per year. It has been reported that particles of plastic-related products have several negative effects in living organisms, for instance, on the gut, intestine, lung, and liver. Polystyrene induces responses, such as oxidative stress, mitochondrial dysfunction, and inflammation representing a risk factor for the kidneys [4]. In general, microplastics can be ingested by humans and organisms ranging from plankton and fish to birds and mammalians throughout the aquatic environment. Additionally, plastics can absorb or contain several chemical additives acting as vectors for multiple organic pollutants [5,6,7].

Multiple efforts are needed to deal with plastic waste generation and to reduce its presence in the aquatic and soil environments. Actions taken worldwide against plastic generation include new policies on plastic prohibition. For instance, the UK government implemented a plastic packaging tax in 2022, applicable to all packaging plastic (manufactured or imported into the UK) that does not contain at least 30% of recycled plastic [8]. In Spain, the free delivery of plastic bags to consumers at sale points of products was prohibited except for plastic bags made of 70% recycled plastic [9]. In New Jersey (USA), starting 2022, a law will be implemented prohibiting selling or providing single-use plastic carryout bags [10]. In Mexico City, these laws have also been established, where single-use plastic carryout bags and plastic straws are prohibited [11].

Bioplastic production emerges as a sustainable alternative to reduce the use of fossil-based plastics and the required degradation time. It is possible to obtain polylactic acid (PLA), polyhydroxyalkanoates (PHAs), and polyhydroxybutyrate (PHB) using biological feedstocks [12,13,14]. Bioplastics have been formulated from various sources, primarily corn, wheat, soy proteins, milk proteins, collagen, and gelatin. However, there are concerns about the long-term viability of these feedstocks, as they compete for land and water resources for human use [15,16]. Microalgae, including cyanobacteria, are a source of renewable biomass. They have gained importance because of their potential use in bioplastic production. Specifically, it is possible to use the entire microalgae, or the polymers synthesized by the cells (i.e., cellulose, starch, PHA, proteins) for bioplastic production [17,18]. Microalgae and cyanobacteria can also be used for wastewater treatment, and the biomass obtained from these processes can be used as feedstock for bioplastic production. 

## 2. Phycoremediation of Wastewater

Microalgae species belonging to different genera, such as *Chlorella* and *Scenedesmus,* have been reported to treat wastewater effluents by assimilation of micro- and macronutrients and adsorption of organic and nonorganic pollutants [19,20]. Table S1 (Supplementary Materials) compiles the most recent advances in the phycoremediation of different wastewaters. *Chlorella* species are recognized for their metabolic plasticity. They can grow fast in wild environment conditions and can exhibit heterotrophic, photoautotrophic, or mixotrophic growth according to the medium requirements [21]. Diverse studies have evaluated the potential of *Chlorella* in wastewater treatment (WWT), in either synthetic or raw wastewater. For instance, synthetic textile wastewater was remediated by *Chlorella vulgaris*, and after 13 days of cultivation, 99% of methylene blue was degraded [22]. These studies have in common that the chemical oxygen demand (COD), nitrogen, and phosphorus removal reached about 70–95%. The removal of pollutants by *Chlorella* species and their feasibility in producing biomass using wastewater as a nutrient source make them a promising candidate for establishing a large-scale wastewater phycoremediation system.

*Scenedesmus* species are also used for wastewater phycoremediation. They can assimilate nitrogen, phosphorus, organic carbon and reduce COD. Besides that, *Scenedesmus* can survive in low-light and polluted environments, such as wastewater from industrial processes, while showing excellent phycoremediation efficiency [23]. For instance, reports on *Scenedesmus* include olive oil mill effluent [24], palm oil mill effluent [25], domestic wastewater [26], industrial wastewater, and brewery effluent [27]. *Scenedesmus obliquus* was cultivated using municipal wastewater, and 0.88 g L^−1^ of microalgal biomass was reached [28]; however, it obtained a lower biomass production compared with BG11 medium [1.3 g L^−1^]. These studies show the potential of these species to phycoremediate wastewater due to its high removal rate of COD, phosphorus, and nitrogen. These results show a promising adaptability of this microalgae to different types of wastewaters.

**Figure 1:**
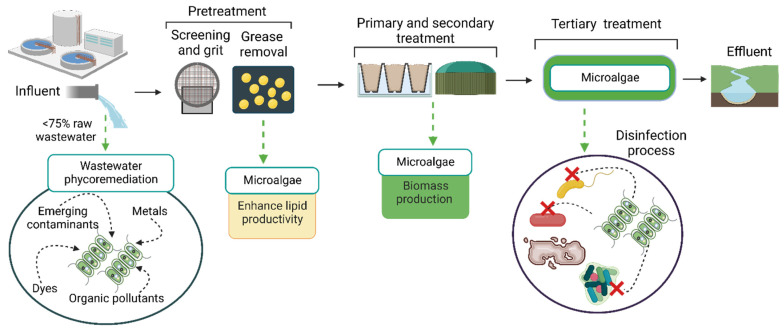
Overview of the wastewater phycoremediation process. Created with BioRender.com (accessed on 10 September 2022).

Phycoremediation of wastewater can be implemented in actual wastewater treatment plants in different steps, such as after a grit and grease removal process, as a primary and secondary wastewater treatment process, and as a third treatment before chlorination, as shown in Figure 1. Few studies are dedicated to studying WWT by microalgae after grit pretreatment. For instance, in a study of Choong et al. (2020) the grease wastewater used as a culture medium enhanced lipid content in *Scenedesmus* and *Tetraselmis* [29]. Additionally, *Ochromonas danica* grown with waste grease as a culture medium accumulated intracellular lipids between 48% and 79% w w^−1^ [29,30,31]. Nevertheless, attention should be paid to the wastewater turbidity and pollutant concentration. Additionally, the residence time should be considered when treating wastewater with microalgae. For instance, in López-pacheco et al. [32], when using wastewater after grit and grease removal, microalgae growth occurred at a maximum of 75% of raw wastewater [32]. This is because a high concentration of raw wastewater has high turbidity and organic load that hinders microalgal cell growth. Therefore, microalgae have a higher potential for WWT as secondary and tertiary treatments.

### 2.1. Disinfection Process in Wastewater by Phycoremediation

Stress can play an important role in microalgal production of antibacterial compounds. Some of these antibacterial compounds (e.g., chlorellin, linolenic acid, phycobiliproteins) are secondary metabolites that have been a valuable source in developing new pharmaceuticals, such as antibiotic, anti-inflammatory, and anticancer drugs [33]. The production of these antibacterial agents depends on the microalgae species; for instance, *Chlorella* species demonstrated antibacterial activity against some bacteria (*Vibrio* bacterial strains) [34]. This antibacterial potential of microalgae culture can possibly be associated with microalgae excretion of substances that inhibit the growth of bacterial strains, such as fatty acids. For instance, in a study by Juttner [35], it was shown that microalgae (diatom consortium mainly composed of *Diatoma elongatum*) release fatty acids as a defense mechanism against grazing predators (e.g., *Favella ehrenbergii*) [35]. *Phaeodactylum tricornutum* was also studied to determine this phenomenon. It was found to liberate fatty acids (capric acid, lauric acid, myristoleic acid, and palmitoleic acid) through lipase action after cell lysis [36,37]. Additionally, in *Chlorella* species, a fatty acid (lipophilic substance) has been identified and named chlorellin, which is excreted during the initial phase of culture growth. The liberation of these fatty acids and lipophilic substances is induced by cell lysis of microalgae already damaged by predators or pathogens. These sacrificial cells protect the culture from further damage since they act as signals or precursors that activate downstream systemic defense responses; this mechanism has also been shown in *Phaeodactylum tricornutum* cultures [38].

There are some studies on the antibacterial capacity of microalgae in aquaculture systems. For instance, *Chaetoceros calcitrans* and *Nitzchia* sp. completely inhibited a *Vibrio* population (*Vibrio harveyi*) within 24 h of exposure in tiger shrimp (*Penaeus monodon*) culture. In the same conditions, *Leptolyngbya* sp. (cyanobacteria])also reduced *Vibrio harveyi* population from 10^4^ to 10^1^ CFU mL^−1^ [39]. Therefore, this type of coculture can help reduce bacterial diseases in aquaculture systems and bacterial load in wastewater. Additionally, there are studies reporting the removal of total and fecal coliforms by microalgae. For instance, *Chlorella sorokiniana* removed 68% of total coliforms (log inactivation: 0.76) and 99% of *Escherichia coli* (log inactivation: 2.73) from a mixture of sanitary wastewater and swine manure [40]. In domestic wastewater, *Chlorella* sp. removed 99% of *Pseudomonas aeruginosa* (log inactivation: 2.5), 99% of total coliforms log inactivation: 2.8), 99% of *Enterococci* (log inactivation: 2.6), and 98% of *Escherichia coli* (log inactivation: 2.2) [41]. This remotion of coliforms is related to an increase in pH during the photosynthetic activity. A pH above 9 is no longer optimal for aerobic and facultative bacteria activity. During cultivation, H^+^ is consumed during the conversion of bicarbonate into CO_2_, and the produced hydroxyl ions accumulate in the medium, causing an increase in the pH and inactivating by this way coliforms; this process is one of the major mechanisms for fecal bacteria remotion in microalgae ponds [42].

Microalgae have also been reported to interact with plants for wastewater phycoremediation. Vetiver-*Dictyosphaerium* sp. c-culture was used in swine wastewater treatment, where about 35 genera of bacteria were detected; of these, 31 genera decreased throughout this treatment process. Specifically, some of the bacteria decreased from approximately 2000 operational taxonomic units (OUT) to zero or near zero (1–228 OUT) (e.g., *Methanosaeta*, *Escherichia*, *Paenibacillus*, *Rhodococcus*, *Ralstonia*, and *Citrobacter*). Additionally, *Escherichia* spp. was completely removed by day 15 of wastewater treatment [43]. 

### 2.2. Biomass Harvesting

Biomass harvesting is a unique process to consider in microalgae production. In this way, there are different techniques to recover as much biomass as possible from the culture system. These methods include flocculation, flotation, centrifugation, and filtration. The harvesting process may depend on the biomass application and culture scale. For instance, when using membrane filtration, biomass of good quality with no chemicals is generated [44]. This is similar to centrifugation, where biomass of good quality is obtained and has a high recovery efficiency (>90%). On the other hand, flocculation can be considered a lower-cost alternative. In fact, flocculation has been used for microalgae harvesting on a large scale and is a common harvesting method in conventional wastewater treatment [45,46]. 

## 3. Sustaining the Unsustainable Products

**Figure 2:**
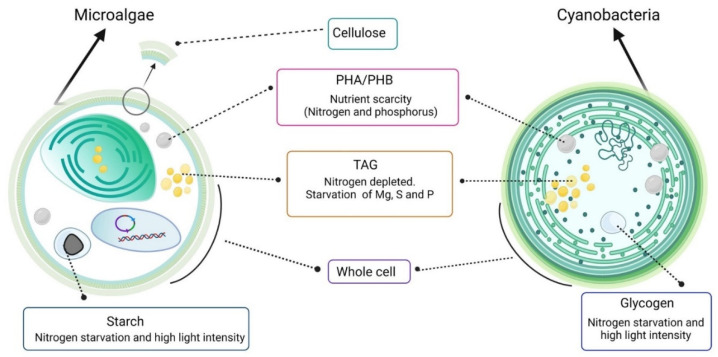
Microalgae and cyanobacteria components used for bioplastic production. PHA: polyhydroxyalkanoates, PHB: polyhydroxybutyrate, TAG: triacylglycerol. Created with BioRender.com (accessed on 10 September 2022).

Microalgae and cyanobacteria are photosynthetic microorganisms that can produce bioplastics, such as polyhydroxyalkanoates (PHAs). Additionally, both can accumulate polymers that can be used as feedstocks for bioplastic. In this section, these polymers are further discussed, including glucose polymers (starch and glycogen) and triacylglycerides (TAGs) (Figure 2). In addition, the possibility of using microalgae and cyanobacteria for bioplastic production by using the whole biomass without fractionation is discussed.

### 3.1. Biomass

Microalgae and cyanobacteria biomass without further fractionation can be used in bioplastic production (as an additive and as a main source). This is mostly because of the carbohydrate content in the biomass (using starch and glycogen in whole cell as a raw material for bioplastic production). For instance, thermoplastic corn starch films mixed with microalgae biomass (*Nannochloropsis gaditana* and *Scenedesmus*) have been reported. The results showed that the addition of microalgae biomass to the thermoplastic corn starch films did not affect the rigidity of the bioplastic [47,48]. In another study, the addition of *Heterochlorella luteoviridis* and *Dunaliella tertiolecta* biomass to cassava starch films increased their solubility, biodegradability, and opacity. Additionally, the produced films showed high antioxidant activity, low water vapor permeability, and good mechanical characteristics, which make them suitable for food packaging. In fact, the authors proved the use of the films for salmon packaging [49].

Compression molding is the most popular method for producing microalgae–polymer blends. By this method, biomass, polymers, and additives are mixed, placed in a mold, and crushed at increased pressure and temperature for a short time to generate bioplastic. In the existing literature, the temperature, pressure, and time parameters differ substantially: temperatures range between 130 and 160 °C with compression pressures ranging from 20 kPa to 10 MPa and molding times ranging from 3 to 20 min [50]. For instance, *Chlamydomonas reinhardtii* biomass was plasticized with glycerol at 120 °C using twin-screw extrusion (processing tool for plasticizing polymers to obtain homogeneous blends), and the bioplastic obtained had a homogeneous plasticized macrostructure [51]. In another study, *Chlamydomonas reinhardtii* biomass was used to produce crude bioplastic beads (7 mm). The biomass was mixed with glycerol or ammonium persulfate and was autoclaved (121 °C for 20 min) and manually molded into beads. These beads were stable in water for at least 7 days and endured compressive strength to 1.7 MPa [52]. Thus, based on these results, it is considered that the production and use of bioplastics from microalgae biomass is feasible. 

### 3.2. Biopolymers

#### 3.2.1. PHA and PHB

Polyhydroxyalkanoates (PHAs) are biodegradable polyesters synthesized by microalgae and cyanobacteria, such as PHB (polyhydroxybutyrate), PHBV (polyhydroxyvalerate), P (4HV) (Poly (4-hydroxyvalerate)), P (3HB-co-3HV) (poly (3-hydroxybutyrate-co-3-hydroxyvalerate)), and P (3HB-co-4HB) (poly (3-hydroxybutyrate-co-4-hydroxybutyrate)) [53]. PHAs produced by microalgae and cyanobacteria have been considered a good substitute for petroleum-based plastics because of their similar mechanical properties [54,55]. The presence of PHB molecules in microalgae cell makes the microalgae-based polymers biodegradable in nature [56]. According to Madadi et al. [57], PHAs have been found in the range of 1–25% dry weight in some cyanobacteria [57]. For instance, PHA was 3.3% in *Synechocystis* sp. [58], 7.4% in *Spirulina* sp. [59], 14% in *Oscillatoria* sp. [60], 21% in *Nostoc* sp. [61], and 25% in *Calothrix* sp. [62], making these species potential candidates for PHA production.

PHAs appear to be generated by microorganisms in response to physiological stress caused by nutrient scarcity. Microorganisms use this polymer as an energy storage molecule that can be digested when other energy sources are unavailable [63,64]. PHA production by microalgae and cyanobacteria can be enhanced by controlling the amount of phosphorus and nitrogen availability in the culture medium. When microalgae and cyanobacteria are deprived of nitrogen, their metabolic process diverts protein to the production of polymers, including starch (or glycogen in the case of cyanobacteria) and PHAs [65]. For instance, *Arthrospira platensis* produced 5.8 mg PHB g^−1^ when deprived of nitrogen [66]. Mourão et al. [67] reported that *Stigeoclonium* sp. was able to produce PHB under nitrogen deprivation and with limited amounts of sodium acetate and sodium bicarbonate [67]. Phosphorus also can be a limiting factor to PHB production [68]. In the case of phosphorus deprivation, microalgae have also shown an increase in PHA production. For instance, *Scenedesmus* sp. produced 29% w w^−1^ of PHA in phosphorus-deprived conditions [69].

The PHA granules from microalgae can be extracted by cell disruption by chemical (sodium hypochlorite), physical (ultrasound and homogenizer mills), and biological methods (use of some enzymes, such as lysozymes, protease, and nucleases). For instance, the use of some solvents, such as 1-butanol, generates a separation process through gelation of PHAs when the mixture is cooled down. Then the solvent can be separated with a rotary evaporator, leaving only the PHAs extracted [54,70,71].

The PHAs obtained from microalgae has a good plasticizing capacity and biodegradability. For instance, *Chlorella* sp., *Oscillatoria salina*, *Leptolyngbya valderiana*, and *Synechococcus elongatus* were used for PHA production under photoautotrophic culture conditions [72]. The PHAs were extracted with sodium hypochlorite (solvent extraction method) and had a glass transition temperature ranging from 4 to 10 °C and a melting temperature ranging between 79 and 116 °C [72]. These characteristics would allow the use of PHAs in the production of bioplastics for medical, agricultural, industrial, food packing, and storage of materials [73]. This leads to considering that the PHAs obtained from microalgae and cyanobacteria are a feasible feedstock for bioplastic production.

#### 3.2.2. Starch and Glycogen

Starch is the major energy reservoir in microalgae and glycogen of cyanobacteria. Both glycogen and starch are glucose polymers accumulated during photosynthesis and can reach about 50% biomass dry weight [74]. Studies have reported that these polymers show very similar characteristics (size of starch granules, amylose/amylopectin content, swelling power, solubility, and turbidity) with commercial corn starch [74]. Thus, starch and glycogen from microalgae and cyanobacteria can also be used for bioplastic production, and they are easily biodegradable [75]. Starch levels in microalgae and cyanobacteria vary significantly. For instance, the red microalga *Porphyridium marinum* has been reported to have 5% biomass dry weight [76], while higher levels of starch content have been reported in other species, such as 6–13% dry weight in *Chlorella* species, 7–18% in *Parachlorella*, and up to 62% in *Tetraselmis* [77,78]. In the case of cyanobacteria, glycogen content has been reported have a 12–24% dry weight in *Synechocystis* sp. PCC 6803 [79,80] and a 27% dry weight in *Anabaena variabilis* [81].

Starch and glycogen accumulation can be enhanced during stress conditions, including nutrient starvation and high light intensity. For instance, *Chlorella* has been reported to increase its starch concentration when is cultivated in a sulfur- and nitrogen-deprived medium [82]. *Chlamydomonas reinhardtii* accumulated 49% w w^−1^ of starch after 20 days of culture with sulfur deprivation, resulting in a starch concentration of 5 g L^−1^ [51]. Starch content in *Chlorella sorokiniana* reached 38% w w^−1^ (0.17 kg m^−3^ day^−1^) when cultivated under high light intensity (300 μmol m^−^^2^ s^−1^) and low nitrogen concentration (32 mg L^−1^) [83]. In the case of cyanobacteria, high light intensity (600 µmol photons m^−2^ s^−1^) was reported to induce up to 31% w w^−1^ glycogen accumulation in *Arthrospira* compared to cultures grown under low light intensity (50 µmol photons m^−2^ s^−1^) in which the glycogen content was 9.4% w w^−1^ [84]. Nitrogen starvation also induces the accumulation of glycogen in cyanobacteria. For instance, Depraetere et al. [85] reported 74% dry weight of carbohydrates, of which about 80 % was identified as glycogen [85]. Similarly, in a study of Hasunuma et al. [86] *Synechocystis* and *Arthrospira* reached about 40% and 60% dry weigh of glycogen during nitrogen starvation [86].

For bioplastic production, starch and glycogen can be extracted from the cells by ultrasonication, bead-beating, and physicochemical methods, such as alkaline and acid hydrolysis with NaOH, HCl, and H_2_SO_4_ [87,88]. For instance, about 90% of the starch in biomass of *Chlamydomonas fasciata* was recovered after 30 min of ultrasonic treatment [89]. In the case of *Nannochloropsis gaditana*, surfactant (Triton X-100)-aided sonication (physicochemical method) was proved to be the most efficient for cell disruption compared with the ultrasonication method [47]. In the case of glycogen, mechanical disruption, such as bead beating, thermolysis (boiled at 100 °C for 40 min), and alkaline hydrolysis (using KOH), are commonly reported [90].

Molding processes, including extrusion, can be applied to produce starch/glycogen–based bioplastics. In this process, glycerol can be added to reduce starch degradation during shear stress and as a plasticizer [91,92]. Gelatinization, compression molding, and the foaming process can also be applied for starch/glycogen processing [93]. Finally, some compounds can be added to starch/glycogen to optimize the bioplastic properties [94]. These compounds include cellulose and laver flack (fiber), which have been reported to improve the mechanical properties of the bioplastic and to reduce the gas permeability, making this material useful for food packing [95].

#### 3.2.3. Cellulose

Cellulose is an appealing choice for bioplastic production due to its broad availability and biodegradability [96]. Cellulose can be found in virtually all photosynthetic species, including plants, seaweeds, tunicates, bacteria, and microalgae. Furthermore, cellulose fibers can be manipulated at the nanoscale, which may generate sophisticated cellulosic biomaterials [97]. The variety and development of cellulose-synthesizing terminal complexes in microorganisms play an important role in the process of industrialization. Cellulose synthesis is carried out by membrane-bound cellulose synthase terminal complexes (related to size, crystallinity, and shape of cellulose microfibril arrangement), containing cellulose synthases [98]. Cellulose in microalgae is found in the cell wall, and it has a different geometry (hexagonal, rosette, single and multiple rows arrangement) that depends on the microalgae taxa [97]. The cell wall in microalgae represents 2% to 10% of the biomass dry weight [99]. The cell wall of *Nannochloropsis* sp. is 75% cellulose (25% of the dry weight) [100,101], 22–25% hemicellulose in *Chlorella vulgaris*, and 23% hemicellulose in *Kirchneriella lunaris* [102]. The cyanobacteria cell wall resembles that of Gram-negative bacteria, which lack cellulose. However, in recent studies, cellulose and cellulose-like structures have been detected in the cell wall of a few species, including *Synechococcus*, *Nostoc*, and *Oscillatoria* [103], making these species good candidates for cellulose extraction.

Cellulose can be used as an additive of starch/glycogen-based bioplastics or polylactic-acid-based bioplastics to improve the bioplastic mechanical properties, such as tensile modulus [104]. After starch is gelatinized by the addition of water and glycerol, cellulose can be added to the mixture. Then, the mixture is casted on acrylic plates and air-dried. The films obtained from this process can be pelletized prior to further use [105]. Hydrogels can also be formed from cellulose [106]. These hydrogels could be formed with LiOH/urea aqueous solution and are further dried (e.g., at 190 °C and 0.1 MPa). Then, cellulose-based bioplastic sheets can be obtained after applying pressure (e.g., 60 MPa at 90 to 190 °C) [107]. The acetylation process is a chemical reaction where the number of hydroxyl interactions is reduced by introducing an acetyl functional group (CH_3_CO) into an organic chemical compound (e.g., cellulose) [108,109]. For instance, cellulose acetate can be formed by adding polyethylene glycol 600 as a plasticizer during the acetylation process [110]. Compared with other polymers, cellulose is not a major fraction in microalgae. However, other compounds of interest, such as pigments, lipids, or starch, can be parallelly extracted besides cellulose, making use of the whole biomass [111].

### 3.3. Miscellaneous Products

#### 3.3.1. Triacylglycerol

Triacylglycerols (TAGs) can be used for bioplastic production; they are derived from a glycerol molecule and three fatty acids. TAGs are the main lipid class in some microalgae species, approximately from 65% to 91%, such as in the case of *Chlorella pyrenoidosa*, *Chlorella vulgaris*, *Phaeodactylum tricornutum*, *Isochrysis galbana*, *Nannochloropsis salina*, and *Scenedesmus* sp. [112]. Nutrient stress, such as nitrogen starvation, is known to promote the production of TAGs. In these conditions, *Chlorella vulgaris*, *Chlorella zofingiensis*, *Neochloris oleoabundans*, and *Scenedesmus obliquus* are reported to accumulate 30–40 w w^−1^ of TAG with a productivity of up to 300 mg TAG L^−1^ day^−1^ [113]. Starvation of other elements, including magnesium, sulfur, and phosphorus, is known to promote TAG production in microalgae [114].

The TAG extraction is usually performed by chemical methods. Some solvents (e.g., methanol, chloroform, hexane, dichloroethane, N-ethylbutylamine, and butanol) are reported as solvents for the extraction of TAGs from microalgal dry biomass. Solvent extraction can recover 69–96% of the total lipids [115,116]. Additionally, the TAG extraction can be performed by ultrafiltration in a single step, using a membrane of regenerated cellulose with molecular weight cutoff of 30 kDa [117]. Additionally, TAG can be recovered and fractionate (mono- and polyunsaturated TAG) by supercritical carbon dioxide extraction; this technology can be scalable at the industrial level [118]. Plastic films from Soybean TAGs were reported by Yu et al. [119]. In their work, the TAGs were dehydrated (60 °C) and mixed with glycerol as a plasticizer at 120 °C. After, the mixture was dried at 110 °C for a week to obtain the plastic films [119].

#### 3.3.2. PLA

**Figure 3:**
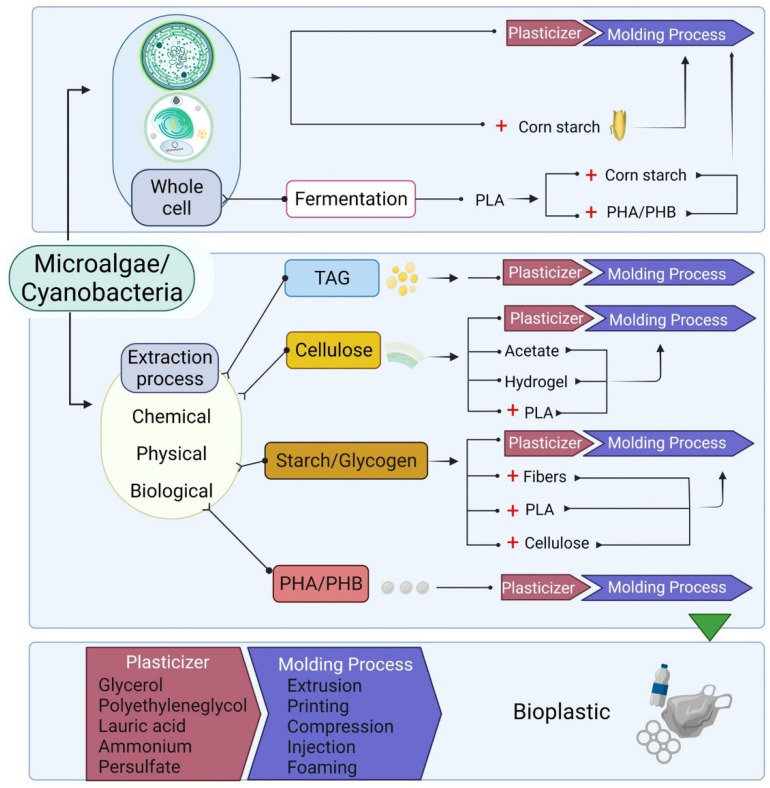
General process of bioplastic production from microalgae biomass. (+: represents a mixing process). Created with BioRender.com (accessed on 10 September 2022).

Polylactic acid (PLA) can be produced by bacteria fermentation of microalgae and cyanobacteria biomass (e.g., *Lactobacillus pentosus* and *Lactobacillus plantarum*) [120,121]. PLA is produced by the polymerization of lactic acid monomers derived from the fermentation of glucose polymers, such as starch and glycogen. PLA can be used usually as food packaging material and electronics products. PLA can be degraded during composting usually after 60 days at 60 °C. Therefore, it has a great potential as a bioplastic due to its high biodegradability [122]. PLA has similar mechanical properties to those of PET, including high tensile strength, hardness, and relatively high elongation [123,124]. PLA production from microalgae biomass has a high productivity; for instance, fermentation of *Chlorella vulgaris* biomass reached a productivity of 9.93 g L^−1^ h^−1^ [120], higher than that reported for cassava bagasse (0.9 g L^−1^ h^−1^) [125], rice straw (2.01 g L^−1^ h^−1^) [126], whey of the dairy industry (2.36 g L^−1^ h^−1^) [127], corn stover (2.32 g L^−1^ h^−1^) [128], and glucose (1.72 g L^−1^ h^−1^) [129]. For bioplastic production from PLA, glycerol can be used as a plasticizer and extrusion at a temperature range of 90 to 150 °C, as reported by Abdullah et al. [130]. Additionally, in the same study, the compression molding process at 150 °C and pressure at 4.9 MPa for 5 min are reported [130]. The bioplastics obtained from PLA can resist water molecules better than bioplastics without this compound, and they can be considered light bioplastics because PLA decreases the density of bioplastics. It has been shown that bioplastic produced with PLA has a lower density than those obtained with other compounds, such as glycerol. Additionally, PHB can be mixed with PLA for bioplastic production [131]. Although there are not many studies about the production of PLA with microalgae and cyanobacteria biomass, these photosynthetic microorganisms are a feasible option to produce PLA bioplastics because of their PLA potential production, the coupling of biomass production processes such as wastewater bioremediation, and lack of lignin. Some of the methods for the bioplastic production with PLA and other compounds obtainable from microalgae biomass can be seen in Figure 3.

## 4. Genetic Engineering to Increase Bioplastic Yield in Microalgae and Cyanobacteria

Genetic engineering is a potential method for modifying the genes of microalgae and cyanobacteria to improve the synthesis of desired polymers, such as starch, TAGs, or PHB [132]. For instance, TAG production in *Neochloris oleoabundans* was increased by co-overexpression of lipogenic genes (plastidial lysophosphatidic acid acyltransferase and endoplasmic-reticulum-located diacylglycerol acyltransferase 2). With this transformation, *Neochloris oleoabundans* increased 1.6-fold the lipid content and 2.1-fold TAG production [133]. The authors also reported a long-term stability of the modified strain since this productivity was maintained for 4 years.

The increase in PHB by using genetic engineering tools has also been reported. In order to induce the production of PHB in *Chlamydomonas reinhardtii*, Chaogang et al. [134] utilized two expression vectors containing the *phbB* and *phbC* genes from *Ralstonia eutropha*, both encoding PHB synthase [134]. The presence of PHB granules in the cytoplasm of the transgenic cells resulted in a favorable outcome, producing 6 µg g^−1^ of PHB compared with no PHB production in the wild-type strain. *Synechocystis* sp. PCC6803 also was modified for enhanced PHB production by the overexpression of a heterologous phosphoketolase (*XfpK*) from *Bifidobacterium breve*, which is used as a strategy to improve acetyl-CoA levels. Using this technique, a PHB production of 232 mg L^−1^ (12% w w^−1^) was obtained under nitrogen depletion conditions, greater PHB production from *Synechocystis* sp. without mutation (1.8% w w^−1^) [135]. Additionally, overexpression of the *phaAB* gene in *Synechocystis* sp. PCC 6803 enhanced PHB production, obtaining a PHB concentration of 35% w w^−1^ growth in nitrogen-deprived medium; also, this technique increased acetyl-CoA levels [136].

Most algal transgenics now employ constitutive promoters to express the recombinant gene throughout algal biomass synthesis, which might have a detrimental influence on growth due to the increased metabolic burden or a potential toxicity on the cell [137]. Thus, a preferable technique is to activate the expression of genes near the end of the growth phase by utilizing tightly controlled promoters with a wide dynamic range in conjunction with good codon optimization, boosting the development efficiency and ultimate production of the targeted gene output. Another problem is the urgent need to create effective chloroplast and mitochondria transformation procedures for most useful microalgal species, as these organelles play critical roles in cellular metabolism. Despite the fact that many projects are underway to generate genome, transcriptome, and proteome information for many microalgal species, it is indeed essential to decode the full annotation of genes and the connectivity of biosynthetic processes in order to fully exploit the prospects of microalgae species.

## 5. Bioplastics from Microalgae and Cyanobacteria Grown in Wastewater

The synthesis of bioplastics from biomass of microalgae and cyanobacteria can be complemented with wastewater treatment. This would allow to grow cellular biomass without requiring synthetic culture medium while also treating wastewater [138]. Nevertheless, up to date, there are few studies that have evaluated this approach. In a study by López Rocha et al. [139], blends of microalgae biomass grown in municipal wastewater were prepared with glycerol. The consortium evaluated in the study included *Scenedesmus obliquus*, *Desmodesmus communis*, *Nannochloropsis gaditana*, and *Arthrospira platensis*. Following injection molding of the blends at 140 °C, bioplastic materials were obtained. Further characterization of the bioplastics formed showed that they had a high thermal stability with low water absorption [139]. In another study, *Desmodesmus* sp. and *Tetradesmus* *obliquus* biomass grown in municipal wastewater was also evaluated to produce bioplastics [140]. In that study, microalgae biomass and glycerol were mixed. The results showed that these bioplastics had similar mechanical properties to bioplastics derived from soy and rice proteins [140].

Most studies on wastewater treatment by microalgae and/or cyanobacteria have focused on PHB production. For instance, PHB from *Botryococcus braunii* grown in sewage wastewater obtained a final PHB of 247 mg L^−1^ [141]. *Synechocystis salina* cultivated in digestate from an anaerobic reactor fed with thin stillage was also evaluated for PHB production [142]. Results showed that at the pilot scale (200 L), 4.8% w w^−1^ of PHB accumulated in *Synechocystis*, similar to that in control cultures grown in synthetic medium [142]. Thus, the PHB production by microalgae/cyanobacteria using wastewater as a culture medium could be feasible.

Although not many studies have considered the production of bioplastics from other macromolecules besides PHB from wastewater-grown microalgae and/or cyanobacteria, it is possible discuss their potential based on the carbohydrate (including starch and glycogen) and lipid (TAGs) content reported in the literature. For instance, *Chlorella vulgaris* grown in aquaculture wastewater obtained a cell density of 3.2 g L^−1^ with a high accumulation of carbohydrates (39% w w^−1^) [143]. *Isochrysis galbana* reached a cell density of 3.2 g L^−1^ with an accumulation of 37% w w^−1^ carbohydrates grown in aquaculture wastewater [143], and *Desmodesmus* spp. grown in landfill leachate and municipal wastewater accumulated 41% w w^−1^ of carbohydrates and 20% w w^−1^ of lipids [144]. Additionally, in that study, it was determined that low concentrations of nitrogen enhance starch production of microalgae culture growth in treated wastewater.

*Chlorella* sp. and *Scenedesmus* sp. grown in domestic wastewater were able to achieve cell growth of 1.78 g L^−1^ and accumulated 34 % w w^−1^ of lipids [145]. *Tetraselmis* sp. grown in municipal wastewater achieved 1.57 g L^−1^ of microalgae biomass with 38% w w^−1^ of lipids [146]. Additionally, *Chlorella sorokiniana* accumulated 43% w w^−1^ of lipids when grown in aquaculture wastewater [147]. *Chlorella* sp. grown in swine wastewater (wastewater characterized for having a high organic load) increased lipid production, including triacylglycerols (2.5 higher times compared with standard medium) [148]. In addition, as it was mentioned before, the used grease wastewater as culture medium enhanced lipid content in microalgae biomass [149]; hence, the use of this wastewater for microalgae culture can also increase the production of bioplastics from these cultures. In Table 1, the production of biopolymers from microalgae grown in different types of wastewaters is expressed.

## 6. Environmental Impact of Bioplastics

**Figure 4:**
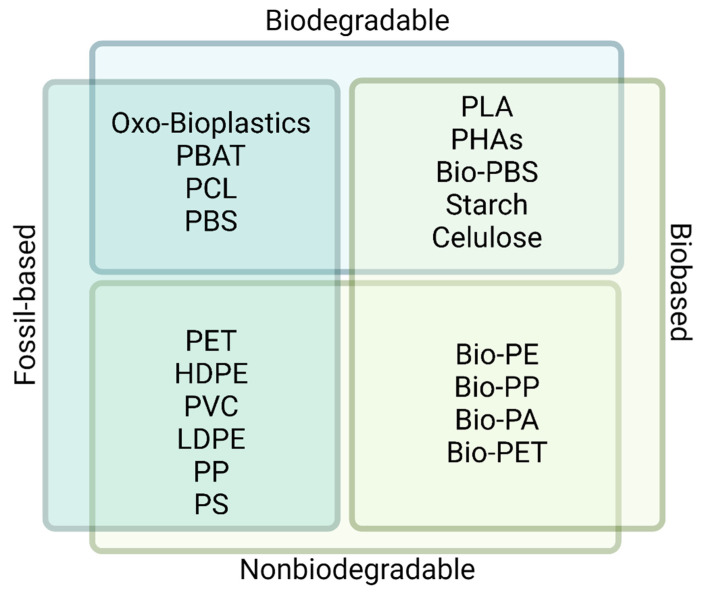
Plastic classification for its biodegradability. Created with BioRender.com (accessed on 10 September 2022).

The environmental impacts of plastics are extremely important and have become a scientific, social, and political issue. Common plastics of petrochemical origin are widely used in different applications due to their low price, durability, and strength. In the last years, derived from their high demand and incorrect disposal, environmental problems have risen due to their accumulation and persistence in terrestrial and aquatic ecosystems. Bioplastics have emerged as an alternative to conventional plastics. They can be produced from materials of biological origin and have a lower impact on the environment [154,155]. Bioplastics are classified in three categories: (1) those that are biobased and biodegradable, (2) fossil-based and biodegradable, and (3) biobased and not biodegradable (Figure 4). Standard plastics (i.e., fossil-based and nonbiodegradable) are not bioplastics. Biodegradable bioplastics can be decomposed by the environment and microorganisms and are thus reintegrated into the ecosystem. For instance, starch-based bioplastics, PLA, and PHA/PHB can be easily degraded in small fragments that are digested by microorganisms. Production of bioplastics is, however, a relatively recent development, and there are still some constrains to be solved, as discussed below.

Land plant crops, such as corn, are currently used for bioplastic production. However, the use of these biomass feedstocks is controversial. They require large areas of cultivation, time, water, fertilizers, and pesticides. These grains are no longer used as food source but in the production of bioplastics and biofuels (e.g., ethanol). In fact, estimations report that a quarter of the cultivated land is currently used to produce biofuels and bioplastics, which has generated a marked increase in the prices of basic foods [156]. Walker and Rothman [156] compared bioplastics from PHB and PLA with plastics of petrochemical origin and determined that the production of bioplastics was more polluting due to the use of fertilizers and pesticides in crops [156]. Nevertheless, Elsawy et al. [157] reported that bioplastics, such as PLA, which are biodegradable and compostable, produce 70% less greenhouse gas emissions during their manufacturing compared with conventional plastics [157]. Therefore, the use of renewable biomass or organic waste can be a strategy to produce ecological bioplastics with lower greenhouse gas emissions [158].

The production of bioplastics using the biomass of microalgae produced from wastewater generates at least two positive impacts on the environment. Microalgae can reduce more than 80% of the nitrogen and COD present in the wastewater; likewise, different types of wastewaters can be used in this process, which demonstrates the versatility of the process [27,28]. Additionally, microalgae could be produced in established wastewater treatment plants, which would not mean the use of arable land for this purpose [159]. On the other hand, the cultivation of microalgae can help reduce the concentration of CO_2_ in the atmosphere, since for every kg of biomass of microalgae produced, 1.8 kg of CO_2_ can be captured in the process [160]. In addition, as stated above, the rate of decomposition of a bioplastic is lower than that of conventional plastics [161], so its use would reduce the production of garbage and reduce the use of land for landfills.

## 7. Economic and Future Perspectives

In 2017, PHA and PLA had prices of USD 5.5 and USD 21 per kilogram, respectively [162]. These prices are considerably high compared with plastics of petrochemical origin (fossil-based plastics), such as polyurethane and polypropylene, with costs of USD 1.7 and USD 1.3 per kilogram, respectively [162]. Although the prices of bioplastics have been reduced to USD 2–6 per kilogram, the prices of conventional plastics of petrochemical origin are still about half, that is, USD 1–2 per kilogram [163]. The cost–benefits of microalgae culture using wastewater culture as a culture medium (the analysis was performed for a flux of 150 m^3^/d of municipal wastewater) were estimated, and it was calculated that the amortization cost (including construction, mechanical, electrical, piping and fittings, and footprint) is approximately USD 249,821. The amortization cost (including construction, mechanical, electrical, piping and fittings, and footprint) is approximately USD 249,821. The operating costs (including reagents, electricity consumption, pumping and mixing, staff, and maintenance costs) were calculated to be USD 29,702 per operation year. The environmental benefits were calculated to be approximately USD 16,885 per operation year, this considering the environmental prices of the undesirable outputs of COD, nitrogen, and phosphorus [28]. Estimating that about 1 g of biomass can be produced every 16 days using wastewater as culture medium, at least 3300 kg of biomass can be obtained per year in the process.

The main limitation of the production and use of bioplastics is the profitability of the process compared with the costs of conventional plastics, which results in more expensive products. Bioplastic production costs can be reduced by exploring and potentiating abundant and low-cost raw materials, such as agricultural, forestry, and food waste; macroalgae and microalgae (including cyanobacteria); technology innovation; and the implementation of a comprehensive biorefinery process based on a circular economy [158,164]. This approach would allow us to increase the profitability of bioplastics and compete with fossil-based plastics.

Bioplastics represent about <1% of the 368 million tons of fossil-based plastic produced annually [165]. Nevertheless, there is an increased demand for bioplastic production and a continuous development of technologies that reduce their production costs. In 2020, 2.11 million tons of bioplastics were produced, and it is estimated that by 2025, 2.87 million tons of bioplastics will be produced. Particularly, 60% of the bioplastics currently produced use PHA, PLA, and starch derivatives as feedstock. Bioplastic production is expected to increase significantly in the coming years associated with new investments and legislation implemented in the USA and Europe [166].

## 8. Conclusions

Bioplastic from microalgae and cyanobacteria biomass can be a great option to reduce the use of petroleum for the production of plastics and also to reduce the decomposition time of these materials. These microorganisms can produce a large amount of biomass in a short time, and compounds such as starch and glycogen, polyhydroxyalkanoates (PHAs), cellulose, polylactic acid (PLA), and triacylglycerols (TAGs) can be used to produce bioplastics. The processes of extraction and processing of bioplastics that have been used up to now have generated bioplastics with optimal characteristics, so it has been proven that it is possible to use these microorganisms for this purpose. Additionally, the production of microalgae and cyanobacteria biomass can be carried out in wastewater, used as a culture medium. In this way, a bioremediation process is carried out and a commercial by-product is obtained. Therefore, by using both processes in combination, an important environmental impact can be generated, helping to solve two major current environmental problems: the treatment of complex wastewater and the plastic pollution crisis.

## Figures and Tables

**Table 1:** Production of biopolymers from microalgae and cyanobacteria grown in wastewater.

Microorganism	Wastewater Type	Polymer Content	References
*Botryococcus braunii*	Sewage wastewater	PHB: 247 mg L^−1^	[141]
*Synechocystis salina*	Digestate from stirred anaerobic tank reactor, fed with thin stillage	PHB: 6.3% w w^−1^ (laboratory-scale) and 4.8% w w^−1^ (pilot scale)	[142]
50% *Scenedesmus obliquus*, 30% *Desmodesmus communis*, 10% *Nannochloropsis gaditana*, 10% *Arthrospira platensis*	Wastewater treatment plant	Dried consortium biomass with glycerol (60 × 10 × 1 mm rectangular-shaped mold)	[139]
*Desmodesmus intermedius*, *Desmodesmus magnus*, *Desmodesmus communis*, *Desmodesmus opoliensis*, and *Tetradesmus obliquus*	Municipal wastewater	Estimated protein in dried biomass (60%) with glycerol (40%) (60 × 10 × 1 mm rectangular-shaped mold)	[140]
*Chlorella* sp. and *Scenedesmus* sp.	Domestic wastewater	Cell density: 1.78 g L^−1^ Lipids: 34% w w^−1^	[145]
*Chlorella* sp.	Seafood processing wastewater	Cell density: 0.89 g L^−1^ Lipids: 28% w w^−1^	[150]
*Chlorella vulgaris*	Aquaculture wastewater	Cell density: 3.2 g L^−1^ Proteins: 30% w w^−1^ Carbohydrates: 39% w w^−1^ Lipids: 6% w w^−1^	[143]
*Chlorella sorokiniana*	Swine wastewater	Cell density: 3.3 g L^−1^ Proteins: 59% w w^−1^ Carbohydrates: 23% w w^−1^ Lipids: 3% w w^−1^	[149,150,151,152]
*Chlorella sorokiniana*	Aquaculture wastewater	Cell density: 0.16 g L^−1^ Lipids: 43% w w^−1^	[147]
*Chlorella pyrenoidosa*	Tofu whey wastewater	Cell density: 2 g L^−1^ Proteins: 34% w w^−1^ Lipids: 17% w w^−1^	[153]
*Scenedesmus obliquus*	Aquaculture wastewater	Cell density: 2.2 g L^−1^ Proteins: 35% w w^−1^ Carbohydrates: 30% w w^−1^ Lipids: 8% w w^−1^	[143]
*Parachlorella kessleri*	Municipal wastewater	Cell density: 1.25 g L^−1^ Lipids: 4.3% w w^−1^	[146]
*Tetraselmis* sp.	Municipal wastewater	Cell density: 1.57 g L^−1^ Lipids: 38% w w^−1^	[146]

## Supplementary Materials

The following supporting information can be downloaded at: https://www.mdpi.com/article/10.3390/md20100601/s1, Table S1: Phycoremediation of wastewater.
