# Supplementary material for: Revalorization of Microalgae Biomass for Synergistic Interaction and Sustainable Applications: Bioplastic Generation
Source: Mar Drugs. 2022 Sep 25;20(10):601. doi: 10.3390/md20100601 (PMC9605595; doi:10.3390/md20100601)
Supplement: Supplementary file 1 [file marinedrugs-20-00601-s001.zip › marinedrugs-1898177-supplementary.pdf]

Supplementary materials

Table S1. Phycoremediation of wastewater

Microalgae	Wastewater characteristics (mg L^{-1})				Removal (%)			Reference
	Wastewater type	Nitrogen	Phosphorus	COD	Nitrogen	Phosphorus	COD	
Chlorella vulgaris	Municipal*	63	8.5	NR	60	95	NR	(Sousa et al., 2021)
	Domestic*	250	42	NR	99	95	NR	
	Textile*	247	22	1,200	95	96	99	(Fazal et al., 2021)
	Municipal	69	3	82	95	96	NR	(Molazadeh et al., 2019)
Chlorella sorokinina	Municipal	34	6.1	211	91	97	93	(Yaqoubnejad et al., 2021)
Chlorella sp.	Soybean	519	130	19950	87	99	77	(Song et al., 2020a)
	Soybean	106	23	5320	96	95	78	(Hu et al., 2020)
Chlorella pyrenoidosa	Municipal	10	2.25	702	99	94	87	(Kumari et al., 2021)
	Anaerobic food	302	25	915	88	67	53	(Tan et al., 2021)
Chromochloris zofingiensis	Palm oil mill effluent	376	58	2898	64	86	44	(Fernando et al., 2021)

Coelastrella sp.	Swine	1550	35	NR	62	77	NR	(Li et al., 2020)
Chlamydomonas reinhardtii	Aquaculture	4.6	15	NR	98	33	NR	(Enwereuzoh et al., 2021)
Haematococcus pluvialis	Palm oil mill effluent	376	58	2898	50	69	50	(Fernando et al., 2021)
Scenedesmus sp.	Palm oil mill effluent	99	53	1,504	100	>80	>80	(Mohd Udaiyappan et al., 2021)
	Potato mill effluent	127	11	NR	59	32	93	(Yuan et al., 2021)
	Municipal	42	3	550	>99	>99	NR	(Ling et al., 2019)
	Brewery*	45	7	2100	79	98	85	(Song et al., 2020b)
Scenedesmus dimorphus	Lactic acid*	651	28	12571	96	90	95	(Zhang et al., 2021)
Scenedesmus obliquus	Domestic	65	5.5	250	>99	83	70	(Han et al., 2021)
Desmodesmus sp.	Piggery	296	28	550	98	81	NR	(Wang et al., 2016)
	Cassava	NR	NR	121	NR	NR	92	(Z. Chen et al., 2020)

*Synthetic wastewaters
COD: chemical oxygen demand
NR: data not reported
